# Fatigue Life Data Fusion Method of Different Stress Ratios Based on Strain Energy Density

**DOI:** 10.3390/ma17122982

**Published:** 2024-06-18

**Authors:** Changyin Wang, Jianyao Yao, Xu Zhang, Yulin Wu, Xuyang Liu, Hao Liu, Yiheng Wei, Jianqiang Xin

**Affiliations:** 1 College of Aerospace Engineering, Chongqing University, Chongqing 400044, China; 20176256@cqu.edu.cn (C.W.); 20176236@cqu.edu.cn (X.Z.); wuyulynn@foxmail.com (Y.W.); liuxuyang@cqu.edu.cn (X.L.); liuhaocqu@cqu.edu.cn (H.L.); 202331131075@stu.cqu.edu.cn (Y.W.); 2 Institute for Aero Engine, Tsinghua University, Beijing 100084, China; xinjq@tsinghua.edu.cn

**Keywords:** torsion fatigue, stress ratio, numerical simulation, strain energy density, data fusion

## Abstract

To accurately evaluate the probabilistic characteristics of the fatigue properties of materials with small sample data under different stress ratios, a data fusion method for torsional fatigue life under different stress ratios is proposed based on the energy method. A finite element numerical modeling method is used to calculate the fatigue strain energy density during fatigue damage. Torsional fatigue tests under different stresses and stress ratios are carried out to obtain a database for research. Based on the test data, the $W_{t}$-$N_{f}$ curves under a single stress ratio and different stress ratios are calculated. The reliability of the models is illustrated by the scatter band diagram. More than 85% of points are within ±2 scatter bands, indicating that the fatigue life under different stress ratios can be represented by the same $W_{t}$-$N_{f}$ curve. Furthermore, *P*-$W_{t}$-$N_{f}$ prediction models are established to consider the probability characteristics. According to the homogeneity of the $W_{t}$-$N_{f}$ model under different stress ratios, we can fuse the fatigue life data under different stress ratios and different strain energy densities. This data fusion method can expand the small sample test data and reduce the dispersion of the test data between different stress ratios. Compared with the pre-fusion data, the standard deviations of the post-fusion data are reduced by a maximum of 21.5% for the smooth specimens and 38.5% for the notched specimens. And more accurate *P*-$W_{t}$-$N_{f}$ curves can be obtained to respond to the probabilistic properties of the data.

## 1. Introduction

Fatigue is a phenomenon in which a material is subjected to a certain number of cyclic loads at a stress level lower than the strength limit, and crack sprouting and expansion gradually occur, eventually leading to fracture failure [1,2]. Fatigue damage is one of the most common forms of failure in engineering practice. It is widely found in vehicle axles, gas turbines, aviation engines, and other major equipment [3,4,5,6]. According to statistics, more than 80% of engineering structure damage is caused by fatigue damage [7].

Solving fatigue damage problems in engineering structures requires fatigue test data to obtain large samples of data. It has been found that even if the fatigue test is conducted under the same conditions, the fatigue life obtained from the same material specimens has deviations, and the fatigue life has an unavoidable discrete nature [8,9,10]. The fatigue test has the features of a long cycle time and high cost; it is difficult to obtain large sample data that meet the requirements of traditional statistical methods. Therefore, to reduce the cost, it is essential to obtain an equal amount of large-sample data and a reliable fatigue life prediction model under small-sample conditions.

In recent years, data fusion methods for small sample data to obtain the same amount of large samples have been developed. The standard deviation principle [11] and the Bayes principle [12,13] are widely used in data fusion principles. The principle of standard deviation considers the conversion of standard deviation between different data levels, and the calculation is simple. However, the distribution law for small sample data is simplified. The Bayes principle can be combined with the prior information of the data to analyze the distribution law of the data. When assessing the fusion effect after data fusion, the S-N curve, or P-S-N curve, is mostly used for testing [14]. For a small sample size, it is difficult to determine the prior distribution reasonably, and the calculation is complicated. Researchers have proposed several methods for the fusion of small sample data and the fitting of P-S-N curves. Xie et al. [11] expanded the fatigue life data of aluminum alloys by standard deviation fusion based on the assumption of a lognormal distribution. The S-N and P-S-N curves were calculated, respectively, and the errors of slope and intercept after fusion were within 7%. Liu et al. [15] proposed an improved backward statistical inference method for fitting P-S-N curves, which can not only obtain reliable fatigue life but also fit more conservative P-S-N curves. Chen et al. [12] used Bayesian and hierarchical Bayesian models for fatigue data analysis and found that the root mean square error of the S-N curves plotted by the hierarchical Bayesian model was less than 5%. Klemenc et al. [16] used a two-parameter Weibull distribution to describe the data dispersion and achieve the goal of fast fitting fatigue P-S-N curves. Shimizu et al. [17] introduced the life distribution equation with three-parameter Weibull and lognormal distribution functions for the three parameters of stress-life indices, fatigue limits, and the basic dynamic stress rating, and proposed a method for analyzing the data using P-S-N curve representation for data analysis.

However, the existing data fusion methods mainly focus on the effect of stress level, and there are fewer studies on other working condition parameters (e.g., stress ratio, etc.) in the fatigue test process. Moreover, the existing fusion methods are mainly based on the statistical characteristics of the data and lack the support of physical meaning. So, the energy approach needs to be introduced to assess the physical significance of the parameters. The energy approach is an important method in the field of fatigue research. The process of fatigue is always accompanied by energy changes, and many fatigue behaviors are closely related to the absorption, consumption, and diffusion of energy [18]. This connection makes the description of fatigue through energy have a more clear physical meaning and is better combined with the stress distribution and different working conditions in the actual fatigue process. The development and research of the energy method provide a theoretical basis for data fusion methods based on more working condition parameters. Since Inglis [19] first proposed the study of energy theory, researchers have gradually linked strain energy methods with fatigue damage assessment [20,21,22,23,24,25], life prediction [26,27,28,29,30], consideration of notch factors [31,32,33], and consideration of stress ratio factors [34,35,36].

The stress ratio is a parameter that reflects the loading process in the fatigue test. However, it is difficult to obtain a large sample of data under any stress ratio using a fatigue test. It is necessary to fuse and expand the data on different stress ratios according to the small sample size. The energy method can provide ideas for data fusion under different stress ratios. In their research on the energy method, considering the effect of the stress ratio factor, Kadi and Ellyin [34] constructed a fatigue failure model based on strain energy and investigated the effect of stress ratios on the fatigue performance of fiberglass unidirectional panels. A standard dimensionless single curve was obtained for different combinations of stress ratios. Kujawski et al. [35] studied the effect of stress ratio on fatigue limit based on strain energy function, and the results showed that the effect of stress ratio on fatigue limit can be described in a unified form. Ellyin [36] found that when the fatigue process was analyzed by energy, the results were not sensitive to changes in the stress ratio. So when studying fatigue behavior based on energy, appropriate normalization can be performed for different stress ratios. This conclusion provides a theoretical basis for performing data fusion under different stress ratios.

Since the existing data fusion methods lack research on different stress ratio conditions, this paper innovatively proposes a data fusion method based on strain energy density for fatigue life under different strain energy density classes and different stress ratio conditions. This method is based on the principle of normalizing the stress ratio by the energy method. The method provides ideas for the fusion and extension of small specimen data under different stress ratio conditions. This paper first establishes and validates the numerical modeling and calculation method for torsional fatigue strain energy density. Based on the fatigue life data, the $W_{t}$-$N_{f}$ and P-$W_{t}$-$N_{f}$ curves under a single stress ratio are calculated, respectively, and the normalized fitting models of different stress ratios are established. Finally, based on the verified data fusion method, fatigue data under different stress ratios are fused to expand the fatigue data and reduce the dispersion of the data. The probabilistic characteristics of the fatigue properties of materials are evaluated more accurately by the fused P-$W_{t}$-$N_{f}$ curve.

## 2. Methodology

### 2.1. Strain Energy Density Calculation Method

To calculate the fatigue strain energy density of the material, fit the $W_{t}$-$N_{f}$ curve, and study the relationship of the energy among different stress ratios, this section first introduces the basic principle of strain energy density and the method of finite element numerical modeling to calculate the strain energy density. Finally, it is validated by the data in the reference.

**Figure 1:**
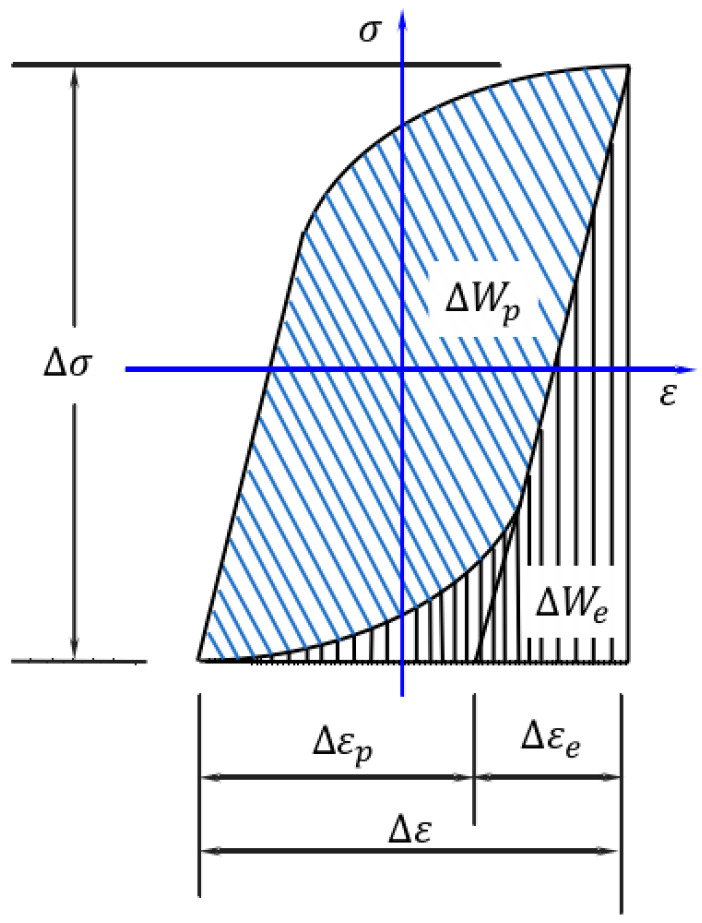
Schematic diagram of strain energy density [39].

In this section, the fatigue process of the material is accompanied by elastic and plastic deformation, and the elastic strain energy densities ${\Delta W}_{e}$ and plastic strain energy densities ${\Delta W}_{p}$ can be calculated by the cyclic stress–strain response curve (the hysteresis loop) in the fatigue process [37,38], and the structure of the hysteresis loop is shown in Figure 1, and the principle of the calculation is expressed in Equations (1) and (2).

(1) $$\Delta W_{e}=\frac{1}{2}\Delta \sigma \Delta \varepsilon -\int_{-\frac{\Delta \sigma }{2}}^{\frac{\Delta \sigma }{2}}\varepsilon d\sigma$$

(2) $$\Delta W_{p}=\int_{-\frac{\Delta \varepsilon }{2}}^{\frac{\Delta \varepsilon }{2}}\sigma d\varepsilon -\int_{-\frac{\Delta \sigma }{2}}^{\frac{\Delta \sigma }{2}}\varepsilon d\sigma$$

where ε and σ represent strain and stress, respectively, and Δε and Δσ represent strain and stress ranges. Based on the Ramberg-Osgood formula [40], the stress–strain relationship under cyclic fatigue load can be expressed by Equation (3):

(3) $$\varepsilon =\varepsilon_{e}+\varepsilon_{p}=\frac{\sigma }{E}+{\left(\frac{\sigma }{K^{\prime }}\right)}^{1/n^{\prime }}$$

where $\varepsilon_{p}$ and $\varepsilon_{e}$ represent plastic strain and elastic strain, E represents elastic modulus, $n^{\prime }$ represents cyclic strain hardening index, and $K^{\prime }$ is cyclic strength coefficient. For materials with Masing characteristics [41], Equations (1) and (2) can be rewritten as Equations (4) and (5):

(4) $$\Delta W_{p}=\frac{1-n^{\prime }}{1+n^{\prime }}\Delta \sigma \Delta \varepsilon_{p}$$

(5) $$\Delta W_{e}=\frac{1}{2}(\Delta \sigma \Delta \varepsilon -\frac{1-n^{\prime }}{1+n^{\prime }}\Delta \sigma \Delta \varepsilon_{p})$$

The elastic strain energy density represented by Equation (4) and the plastic strain energy density represented by Equation (5) are added to obtain the total strain energy density $W_{t}$, as follows:

(6) $$W_{t}=\Delta W_{e}+\Delta W_{p}=\frac{\Delta \sigma \Delta \varepsilon_{p}}{1+n^{\prime }}+\frac{\Delta \sigma \Delta \varepsilon_{e}}{2}$$

**Figure 2:**
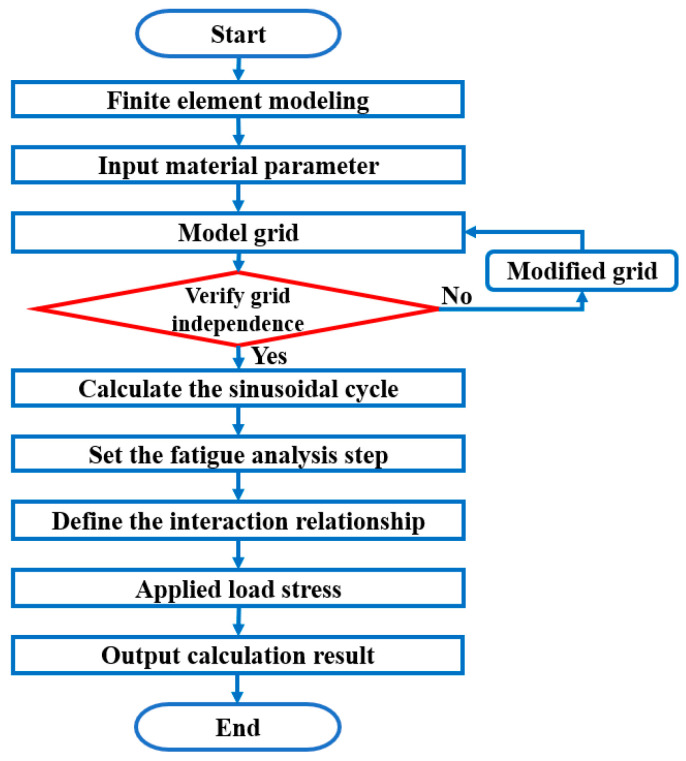
Strain Energy Density Modeling Calculation Flow.

In this paper, numerical simulation modeling is carried out using the finite element software ABAQUS 2019 to calculate the fatigue strain energy density during the fatigue process. The specific modeling and calculation flow are shown in Figure 2:

(1) Finite element modeling based on test specimens;
(2) Inputting the elastic and plastic parameters of the material;
(3) Dividing the model mesh and verifying mesh independence;
(4) Selecting the number of fatigue cycles and calculating the sinusoidal cycle curve based on the number of cycles and stress ratio;
(5) Applying the sinusoidal cyclic load to the specimen and obtaining the calculation results.

The correctness of the numerical modeling approach is verified based on the data in ref. [39]. Based on the numerical modeling procedure in Figure 2, the same tensile fatigue specimens are modeled and calculated. The material constants are shown in Table 1. The simulation results are shown in Table 2. The relative error between the calculated results of this paper and those of the ref. [39] is within ±2.1%. It indicates that the method is correct.

### 2.2. Torsional Fatigue Test Method and Data

To investigate the relationship between strain energy density and torsional fatigue life of a certain type of alloy, torsional fatigue tests are conducted in this section to obtain the data research basis.

**Figure 3:**
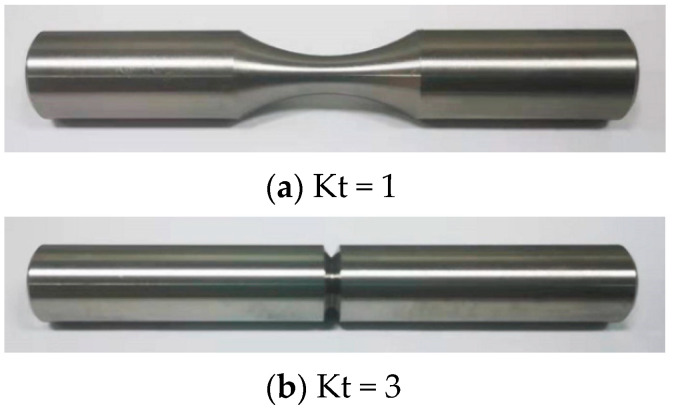
Schematic diagram of a room-temperature specimen.

**Figure 4:**
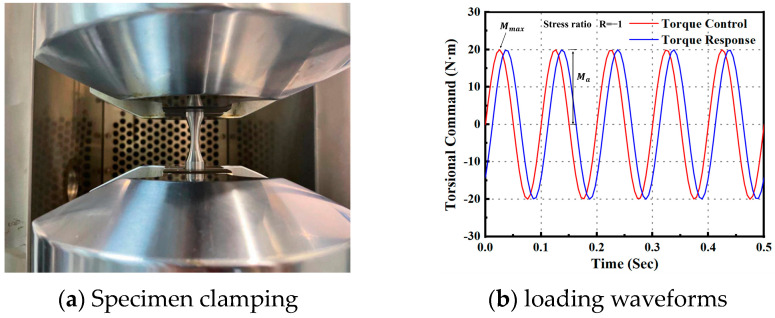
Specimen clamping and loading waveforms.

**Figure 5:**
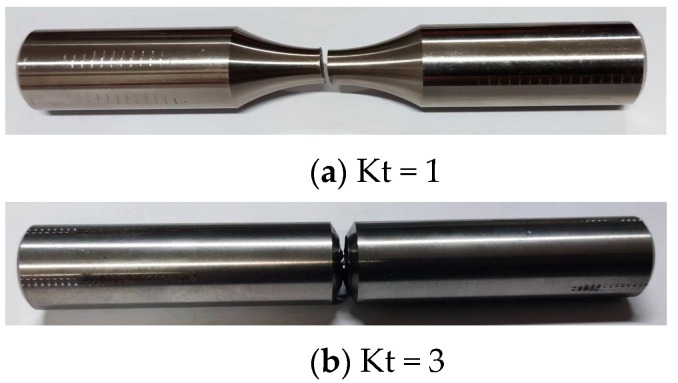
Schematic diagram of the fracture of room-temperature specimens.

**Figure 6:**
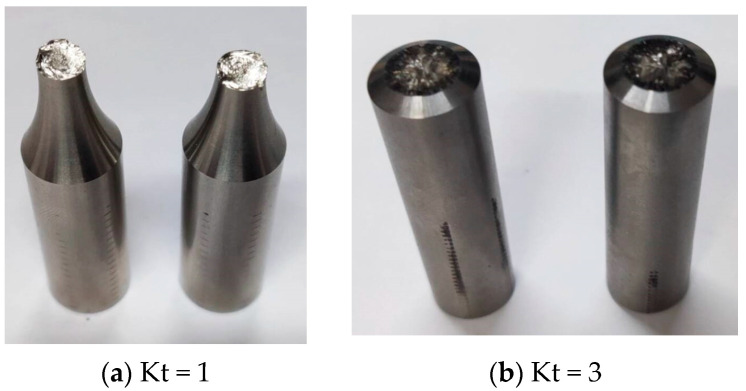
Fracture morphology of room-temperature specimens.

The tests were conducted using the torsional stress fatigue criterion at room temperature (25 ± 5 °C) in air. Smooth (Kt = 1) and notched (Kt = 3) specimens are shown in Figure 3. The specimens were manufactured based on the standards of the Aeronautical Materials Handbook [42]. The specimen was clamped in the fatigue testing machine, as shown in Figure 4a. The lower end of the tester held the specimen in place, and the upper end applied a sinusoidal load with a loading frequency of 10 Hz. The load was applied according to the maximum torsional stress. The maximum stress was adjusted by the grouping method. Stress ratios were selected as −1 and 0.05. With the stress ratio being −1, for example, the torque loading waveform is shown in Figure 4b. Torsional fatigue tests under different stresses and stress ratios were conducted according to the above test conditions. The fracture morphology under smooth and notched specimens after torsional fatigue testing is shown in Figure 5 and Figure 6. The results show that the fracture surface of the smooth specimen has obvious crack initiation and extension zones, and the fracture surface of the notched specimen is relatively rough.

**Figure 7:**
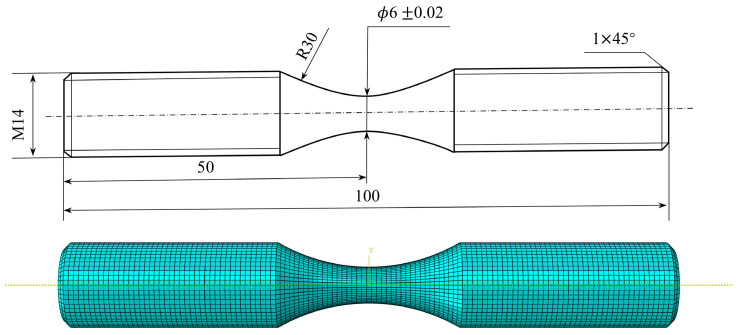
Smooth specimen size parameters and finite element modeling.

**Figure 8:**
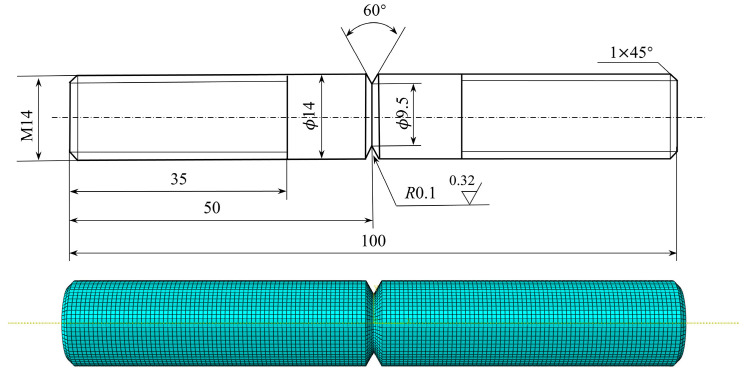
Notched specimen size parameters and finite element modeling.

**Figure 9:**
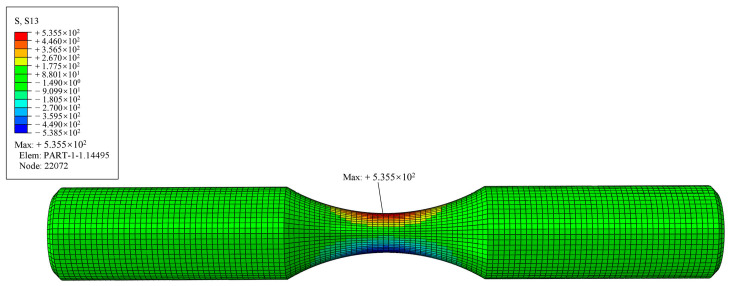
Finite element calculations for size 0.8 mm.

**Figure 10:**
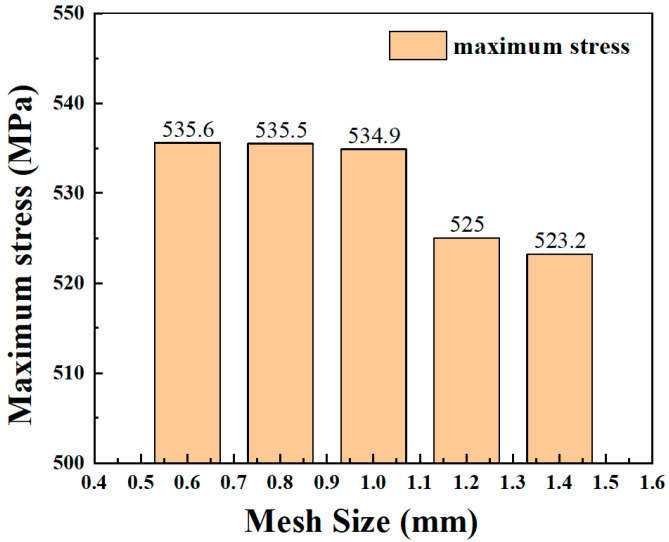
Finite element calculation results for different size dimensions.

**Figure 11:**
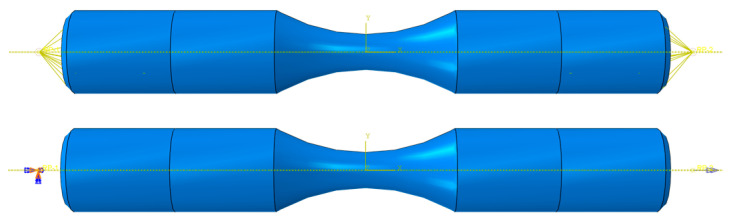
Smooth specimen coupling relationships and loads.

**Figure 12:**
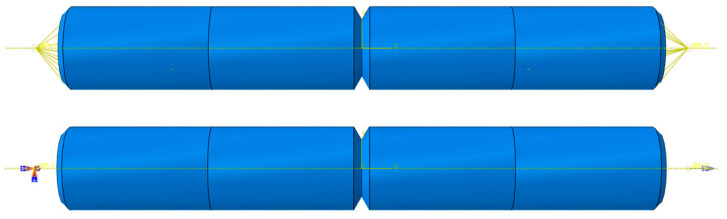
Notched specimen coupling relationships and loads.

Based on the experimental standard working conditions and loading conditions, this section calculates the fatigue strain energy density of each working condition in torsional fatigue. Finite element models were established based on the reference standard specimen size, and the model meshes were divided, as shown in Figure 7 and Figure 8. Table 3 shows the input of the material constant of the finite element model. When the mesh size of the finite element model is 0.8 mm, the maximum torsional stress in the S13 direction of the specimen is 535.5 MPa, as shown in Figure 9. The finite element models with different mesh sizes are calculated and compared, as shown in Figure 10. It is found that the model has converged at a mesh size of 0.8 mm, so the mesh size chosen for modeling in this paper is 0.8 mm. In setting up the interaction, the ends of the specimen are dissected and coupled to reference points to simulate the clamping of the test equipment. When applying the load, one end of the specimen is completely fixed, and the other end is loaded with a sinusoidal alternating concentrated torque load to simulate the loading of the equipment, as shown in Figure 11 and Figure 12. The torsional fatigue data and calculated fatigue strain energy density results are shown in Table 4 and Table 5.

### 2.3. $W_{t}$- $N_{f}$ Curve and P-$W_{t}$-$N_{f}$ Curve Calculation Method

The strain energy density-fatigue life ($W_{t}$-$N_{f}$) curve can be solved using a two-parameter power function model [43] as shown in Equation (7) or a three-parameter power function model [44] as shown in Equation (8). Formally, the three-parameter model has one more constant term than the two-parameter model, which can express the endurance limit of the model curve. The physical meaning of the endurance limit is that the material will not undergo fatigue damage when the strain energy density is below the endurance limit. However, the calculation principle of the three-parameter model is more complicated.

(7) $$W_{t}=\Delta W_{e}+\Delta W_{p}=A{\left(N_{f}\right)}^{B}$$

(8) $$W_{t}=\Delta W_{e}+\Delta W_{p}=A{\left(N_{f}\right)}^{B}+C$$

The probabilistic fatigue strain energy density-fatigue life (*P*-$W_{t}$-$N_{f}$) curve is solved according to the one-side tolerance factor method [15]. Based on the average fatigue life $x_{a}$ and standard deviation σ at each strain energy density, the probabilistic fatigue life $x_{p}$ can be expressed as:

(9) $$x_{p}=x_{a}+k_{\left(p,\gamma ,\nu \right)}\cdot \sigma$$

where $k_{\left(p,\gamma ,\nu \right)}$ is the one-side tolerance factor. p is the survival rate, which is 90% and 99% in this paper. γ is the confidence degree, which is uniformly selected as 95% in this paper. ν is the degree of freedom, depending on the number of samples.

The one-side tolerance factor is related to the number of samples. When the number of samples increases, the value of $k_{\left(p,\gamma ,\nu \right)}$ will decrease, making the standard deviation more accurate. To obtain more accurate *P*-$W_{t}$-$N_{f}$ curves, it is necessary to expand the sample size by using reliable data fusion methods.

### 2.4. Data Fusion Method

**Figure 13:**
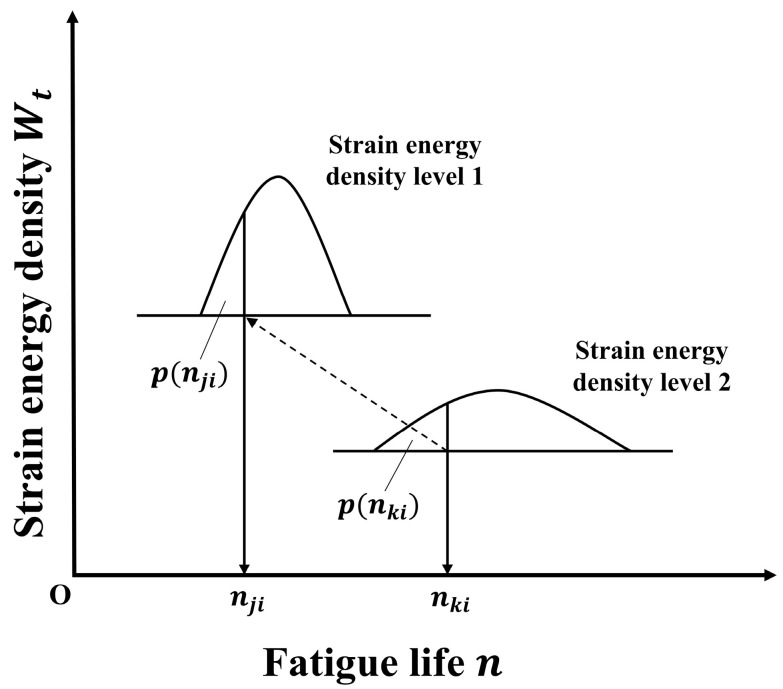
Lifetime probability quantile consistency schematic diagram [10].

In this section, the data fusion method for fatigue life of different stress ratios by energy method is presented, and the specific process is given. The fatigue life data of small samples was fused based on the point consistency principle of fatigue probability proposed in ref. [10]. The fatigue probability loci consistency principle means that no matter how the strain energy density level and stress level of the sample change, the test life of the same specimen at different levels will correspond to the same probability loci of the life distribution determined by the strain energy density level of the sample, as shown in Figure 13. The lifetime probability loci consistency principle can be expressed by Equation (10):

(10) $$p\left(n_{ji}\right)=p(n_{ki})$$

where $n_{ji}$ represents the fatigue life of specimen i at strain energy density level j, $n_{ki}$ represents the fatigue life of specimen i at strain energy density level k, $p\left(n_{ji}\right)$ represents the probability of life being less than $n_{ji}$ at strain energy density level j, and $p(n_{ki})$ represents the probability of life being less than $n_{ki}$ at strain energy density level k.

For the test data subject to a lognormal distribution, the probability distribution is shown in Equation (11). Combined with Equation (10), Equation (12) can be obtained for mutual conversion under different strain energy densities:

(11) $$P\left(N<{lgn}_{i}\right)=\phi (\frac{{lgn}_{i}-\mu_{i}}{\sigma_{i}})$$

(12) $$\frac{lgn_{ji}-\mu_{j}}{\sigma_{j}}=\frac{lgn_{ki}-\mu_{k}}{\sigma_{k}}$$

where $\mu_{j}$ and $\mu_{k}$ represent the mean logarithmic fatigue life at strain energy density levels j and k, respectively, and $\sigma_{j}$ and $\sigma_{k}$ represent the standard deviation of logarithmic fatigue life at strain energy density levels j and k, respectively.

According to the standard deviation fusion method [45], the relationship between the standard deviation of logarithmic fatigue life and strain energy density under equivalently large samples is shown in Equation (13):

(13) $$\sigma_{j}^{\prime }=\sigma_{k}^{\prime }+K(W_{tk}-W_{tj})$$

where $W_{tj}$ and $W_{tk}$ represent the strain energy density levels j and k, respectively, and K is the fitting coefficient, which can be used as the convergence parameter in the fusion process.

**Figure 14:**
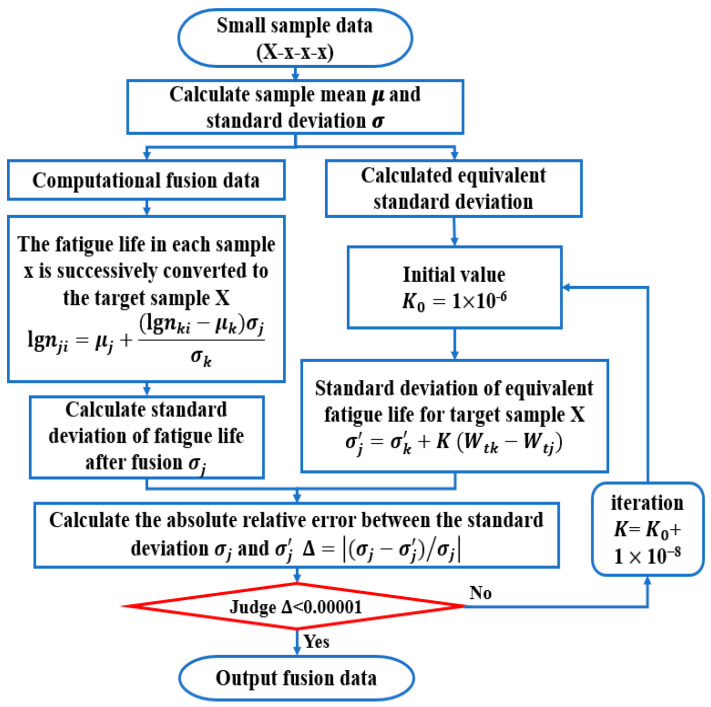
Data fusion flow chart.

Based on the above data fusion theoretical methods, the data fusion process in this section is shown in Figure 14:

(1) Calculating the mean logarithmic fatigue life of each strain energy density level;
(2) Calculating the logarithmic life standard deviation of the fusion target level;
(3) Fusing data from other strain energy density levels and calculating the standard deviation of the fused life data;
(4) Setting an initial value of $K_{0}$ and calculating the equivalent standard deviation from other levels to the target level;
(5) Calculating the relative error Δ of two standard deviations. When Δ meets the error requirement, the fusion result is the output.

## 3. Results and Discussion

### 3.1. $W_{t}$-$N_{f}$ Curve Fitting from Test Data

**Figure 15:**
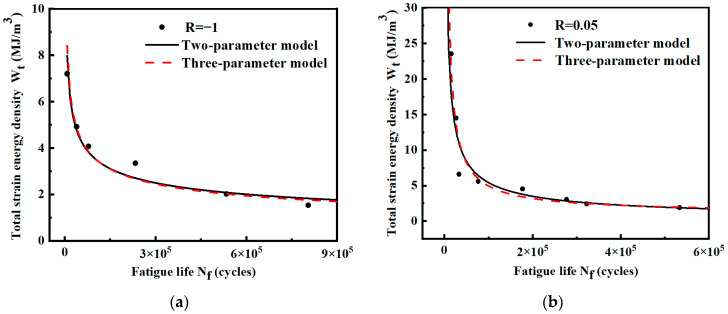
$W_{t}$-$N_{f}$ curves of smooth specimens under different stress ratios. (**a**) Stress ratio R = −1; (**b**) stress ratio R = 0.05.

For the smooth specimen shown in Figure 3, the fatigue life and strain energy density in Table 4 are fitted by two-parameter and three-parameter models, respectively. The power functions are shown in Figure 15. The expressions of the models are shown in Table 6. The results show that the life prediction error of the two models is small under different strain energy densities. Under the condition of R = 0.05, the two-parameter model has a higher R^2^ value and stronger prediction ability.

**Figure 16:**
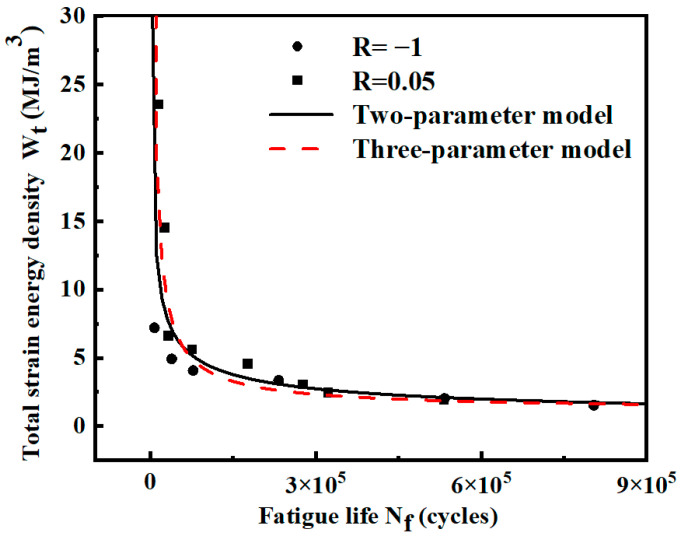
$W_{t}$-$N_{f}$ curves of smooth specimens fitted by two stress ratios.

For the same specimen, the fatigue strain energy density leading to material damage is the same for the same life when the fatigue loading conditions are just different stress ratios. Based on this assumption, the strain energy density and fatigue life data under two different stress ratios are fitted using a two-parameter model and a three-parameter model, respectively. The fitted curves are shown in Figure 16. The expressions of the two models are shown in Table 6. The results show that the R^2^ value of the two-parameter model is still larger than that of the three-parameter mode. Considering that the three-parameter model is more complicated and the R^2^ value is smaller than that of the two-parameter model, only the two-parameter model is used below to analyze and calculate the smooth specimens.

**Figure 17:**
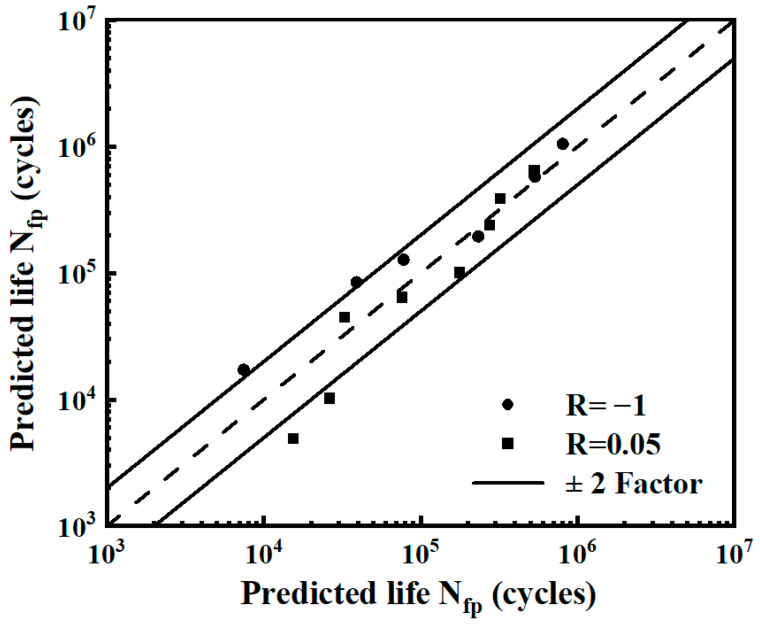
The scatter band diagram of the smooth specimen.

Based on the two-parameter model, the predicted life under different strain energy densities in the fatigue test is calculated. The scatter band diagram between the predicted life and the test life is shown in Figure 17. The results show that more than 85% of points were within ±2 scatter bands, indicating the accuracy of the two-parameter model.

**Figure 18:**
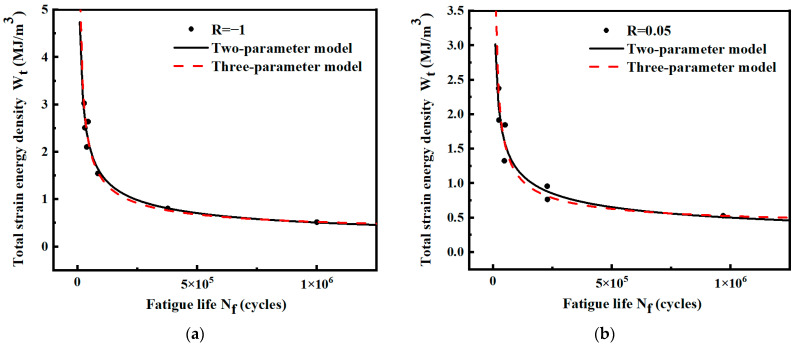
$W_{t}$-$N_{f}$ curves of notched specimens under different stress ratios. (**a**) Stress ratio R = −1; (**b**) stress ratio R = 0.05.

For the notched specimen shown in Figure 3, according to the calculated data in Table 5, the strain energy densities are fitted with the fatigue test life through the two-parameter and three-parameter models, respectively. The power functions are shown in Figure 18. The model expressions are shown in Table 7. The results show that the life prediction error of the two models is small under different strain energy densities. The R^2^ values of the two models are the same under the two stress ratios. Moreover, the fatigue life of notched specimens is smaller and more concentrated under the same strain energy density, the R^2^ value of notched specimens is higher than that of smooth specimens.

**Figure 19:**
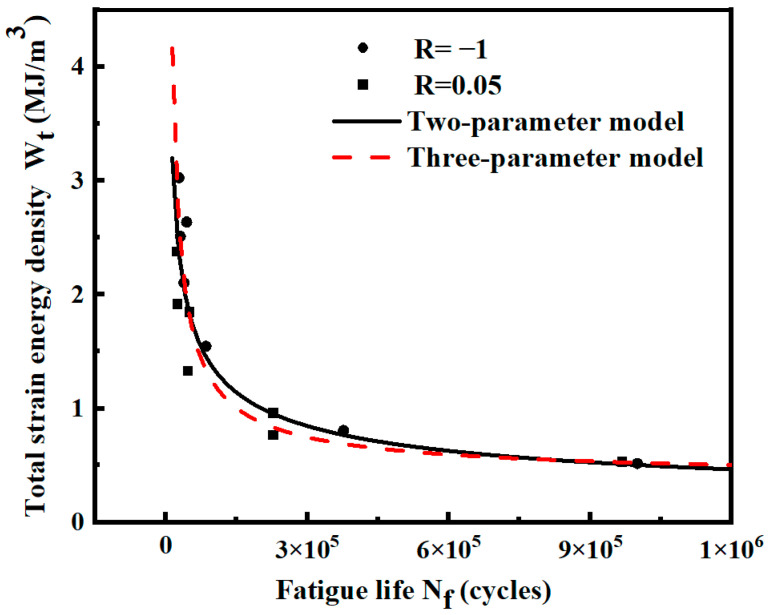
$W_{t}$-$N_{f}$ curves of notched specimens fitted by two stress ratios.

The strain energy density and fatigue life data of the notched specimens under two different stress ratios are summarized. The fitting calculation is conducted by the two-parameter and three-parameter models, as shown in Figure 19. The expressions for both models are shown in Table 7. When the two stress ratios are combined, the R^2^ values of the two-parameter model are higher than those of the three-parameter model. Therefore, considering the high calculation cost and low R^2^ value of the three-parameter model, only the two-parameter model is used to analyze and calculate the notched specimen.

**Figure 20:**
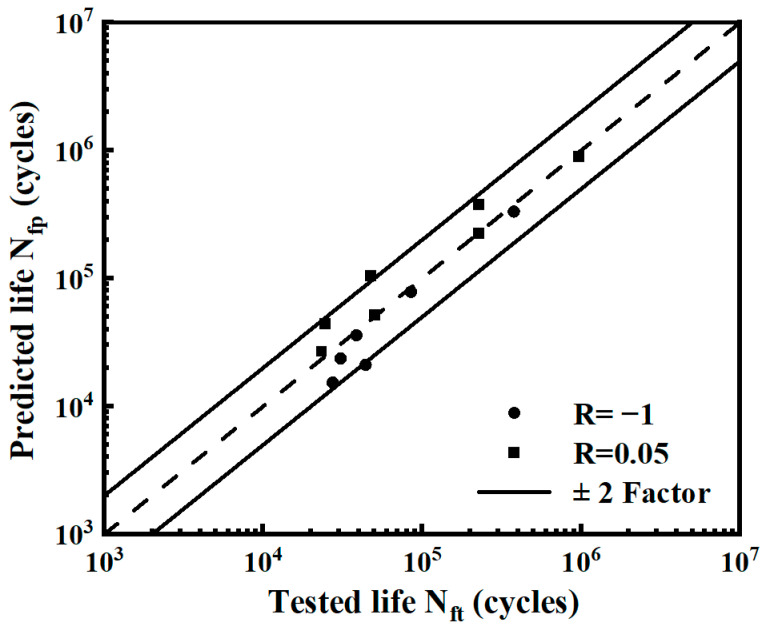
The scatter band diagram of the notched specimen.

Based on the two-parameter model, the predicted life under different strain energy densities in the fatigue test is calculated. The scatter band diagram of the predicted life and the test life is shown in Figure 20. The results show that more than 85% of the points are within the ±2 scatter bands, indicating the accuracy of the two-parameter model.

### 3.2. P-$W_{t}$-$N_{f}$ Curve Fitting

To better describe the dispersion of fatigue test data, it is of engineering significance and reference value to establish fatigue life curves with a specified probability. Using the same treatment method as P-S-N, life can be probabilized based on the obtained $W_{t}$-$N_{f}$ curve.

**Figure 21:**
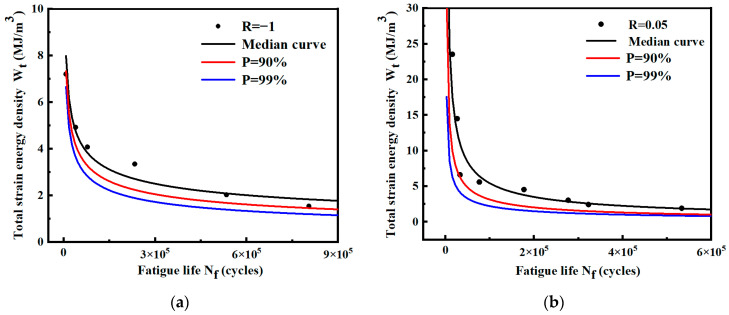
*P*-$W_{t}$-$N_{f}$ curves of smooth specimens under different stress ratios. (**a**) Stress ratio R = −1; (**b**) stress ratio R = 0.05.

**Figure 22:**
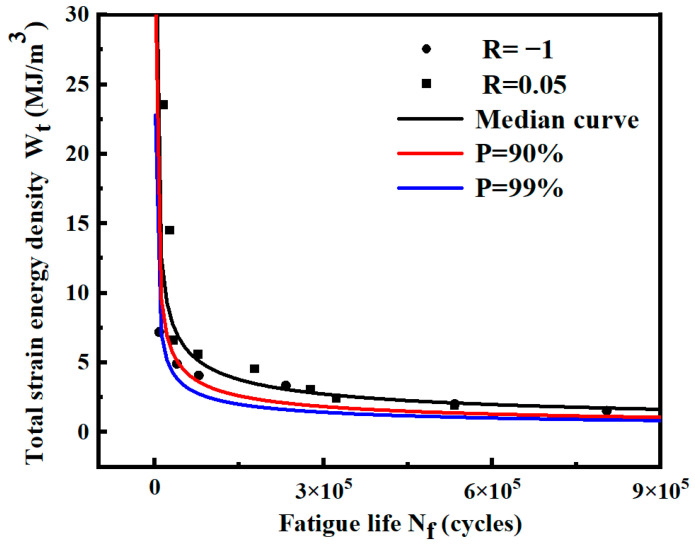
*P*-$W_{t}$-$N_{f}$ curves of smooth specimens fitted by two stress ratios.

The probability fatigue life of smooth specimens under various working conditions is calculated by Equation (9), and *P*-$W_{t}$-$N_{f}$ curves are shown in Figure 21 and Figure 22. The model expressions of different curves are shown in Table 8. The results show that the fatigue life predicted by the *P*-$W_{t}$-$N_{f}$ curve is more conservative. Most of the fatigue data points are above the probability curve of *P* = 90%, indicating that the probability distribution of *P* = 90% can meet the requirements of practical engineering.

**Figure 23:**
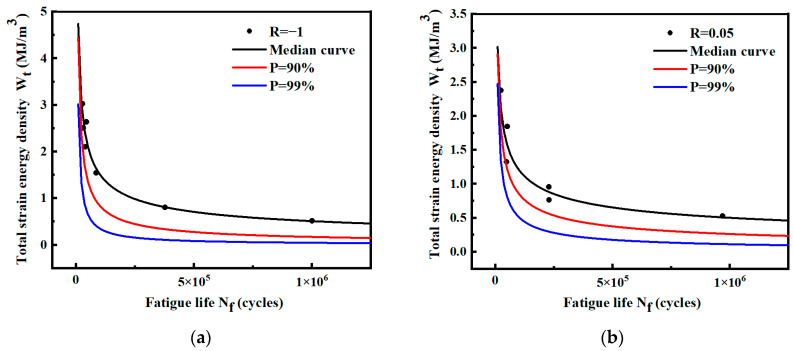
*P*-$W_{t}$-$N_{f}$ curves of notched specimens under different stress ratios. (**a**) Stress ratio R = −1; (**b**) Stress ratio R = 0.05.

**Figure 24:**
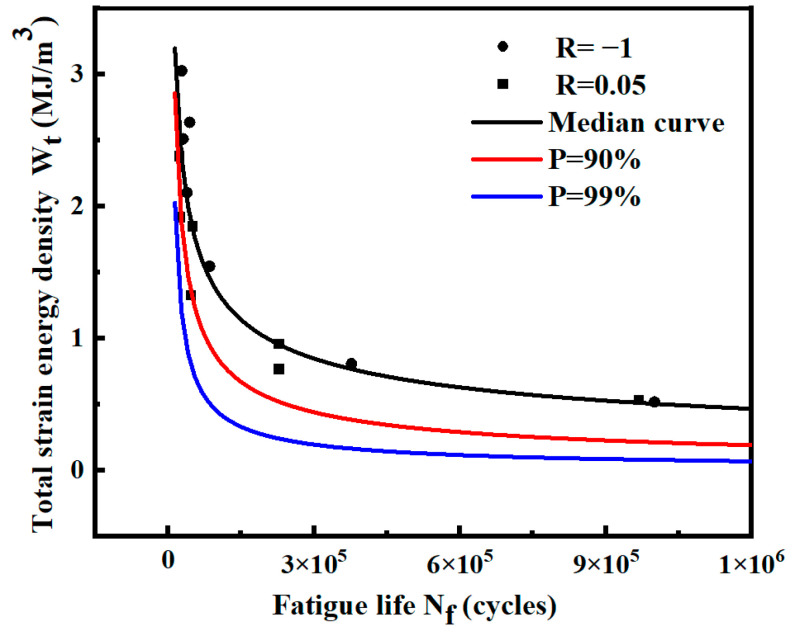
*P*-$W_{t}$-$N_{f}$ curves of notched specimens fitted by two stress ratios.

The probabilistic fatigue life of notched specimens under different conditions is calculated by Equation (9), and the *P*-$W_{t}$-$N_{f}$ curves are shown in Figure 23 and Figure 24. The model expressions of different curves are shown in Table 9. The results show that most fatigue data points are above the probability curve of *P* = 90%, indicating that the requirement can be satisfied by considering the probability distribution of *P* = 90% in practical engineering.

### 3.3. Data Fusion and P-$W_{t}$-$N_{f}$ Curve Fitting Based on the Overall Data

To verify the correctness of the strain energy data fusion method, 600 fatigue test data points from ref. [46] are selected as large sample test data for calculation. According to the bending fatigue test conditions in ref. [46], three strain energy density levels are calculated. A random number program was used to randomly select five fatigue life test data points from each strain energy density level, as shown in Table 10. Table 11 shows the fatigue life data of each strain energy density level obtained using the data fusion method.

The mean and standard deviation of the pre-fusion data, the post-fusion data, and the overall data are shown in Table 12. Comparing the post-fusion data with the overall data, the logarithmic standard deviation of the data at each level after fusion is smaller than that of the overall data of the large sample. The maximum value of the standard deviation reduction is 30.1%. It indicates that the data fusion method can effectively expand the data volume and reduce the data dispersion.

**Figure 25:**
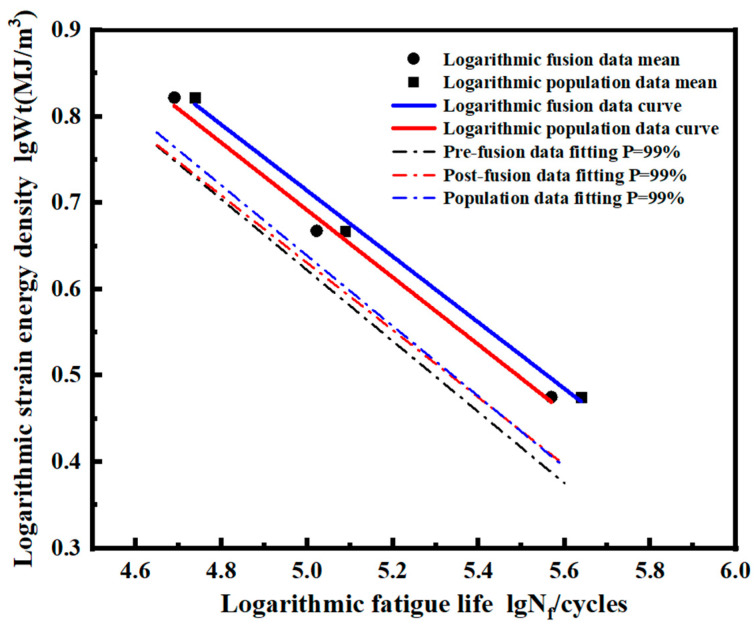
Comparison of logarithmic *P*-$W_{t}$-$N_{f}$ curves of pre-fusion data, post-fusion data, and overall data.

Based on the two-parameter model, the logarithmic median $W_{t}$-$N_{f}$ curve and logarithmic *P*-$W_{t}$-$N_{f}$ curve at *P* = 99% of the pre-fusion data, post-fusion, and the overall data are shown in Figure 25. Only the logarithmic median $W_{t}$-$N_{f}$ curve after fusion is shown in Figure 25 because the mean value of the pre-fusion data and post-fusion data is the same and the logarithmic median $W_{t}$-$N_{f}$ curve coincides. The slope of the logarithmic $W_{t}$-$N_{f}$ curve fitted based on the post-fusion data is −0.390, and the intercept is 2.642. The slope of the logarithmic $W_{t}$-$N_{f}$ curve fitted based on the overall data is −0.382, and the intercept is 2.625. Based on the overall data, the slope relative error of the two curves is 2.08%, and the intercept relative error is 0.65%. For the probability curve of *P* = 99%, the results show that the logarithmic *P*-$W_{t}$-$N_{f}$ curve after fusion at a low strain energy density level is closer to the overall data than that before fusion. This is because data fusion expands the data volume and reduces the dispersion of low-strain energy density data.

**Figure 26:**
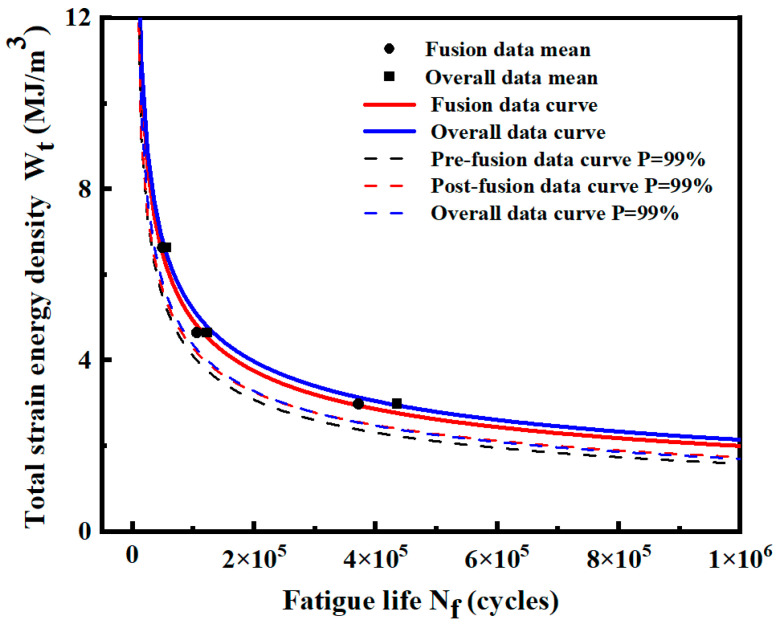
Comparison of *P*-$W_{t}$-$N_{f}$ curves of pre-fusion data, post-fusion data, and overall data.

Figure 26 shows the median $W_{t}$-$N_{f}$ curve and the *P*-$W_{t}$-$N_{f}$ curve at *P* = 99% of pre-fusion, post-fusion, and overall data. The expressions of each fitting curve are shown in Table 13. For the median curve, the position of the fusion curve and the overall data curve are close at the high strain energy density level and gradually deviate at the low strain energy density level. Table 13 shows the predicted life values and relative errors of the two median curves under three different strain energy density levels. Based on the overall data, the minimum relative error between the fusion data and the overall data is 11.48%, and the maximum relative error is 15.19%. The error is due to the inevitable difference between the average value of the selected small samples and the overall data. This error is within the acceptable range.

For the probability curve at *P* = 99%, results show that the *P*-$W_{t}$-$N_{f}$ curve after fusion is closer to the *P*-$W_{t}$-$N_{f}$ curve of the overall data than that before fusion. In Table 13, only at the high strain energy density level (6.639 MJ/m^3^) is the prediction error of 7.85% after fusion larger than that of 6.99% before fusion. At the middle and low strain energy density levels (4.652 and 2.987 MJ/m^3^), the prediction errors after fusion are smaller than those before fusion. Moreover, the prediction ability of the fusion data is improved continuously from a high strain energy density level to a low strain energy density level. The minimum prediction error is 0.87% at a low strain energy density level. The life prediction error between pre-fusion data and overall data is increasing. It shows that the data fusion method can expand the fatigue data at different strain energy density levels and reduce the dispersion of fatigue data at middle and low strain energy density levels. Post-fusion data are closer to the overall data, which verifies the effectiveness of the data fusion method.

### 3.4. Data Fusion and P-$W_{t}$-$N_{f}$ Curve Fitting under Different Stress Ratios

Based on the verification of the hypothesis that stress ratio is irrelevant in strain energy density analysis of fatigue failure in Section 2.2, this section proposes to perform data fusion on logarithmic fatigue life under different stress ratios and strain energy densities. In this way, data can be expanded based on existing small sample data under different strain energy densities and stress ratios. A more accurate *P*-$W_{t}$-$N_{f}$ curve is obtained, which provides a reference value for engineering applications.

For smooth specimens, according to the test data in Table 4, two stress ratios and two strain energy density levels for each stress ratio are selected for data fusion. The pre-fusion data and post-fusion data are shown in Table 14. The comparison of the logarithmic mean and logarithmic standard deviation before and after fusion is shown in Table 15. After the fusion of different stress ratios, the average logarithmic life of each strain energy density level remains unchanged. The standard deviation after fusion at each strain energy density level is smaller. Compared with the pre-fusion data, the maximum reduction in the standard deviation of the post-fusion data is 21.5%. The results show that the data fusion method reduces data dispersion while expanding the data.

**Figure 27:**
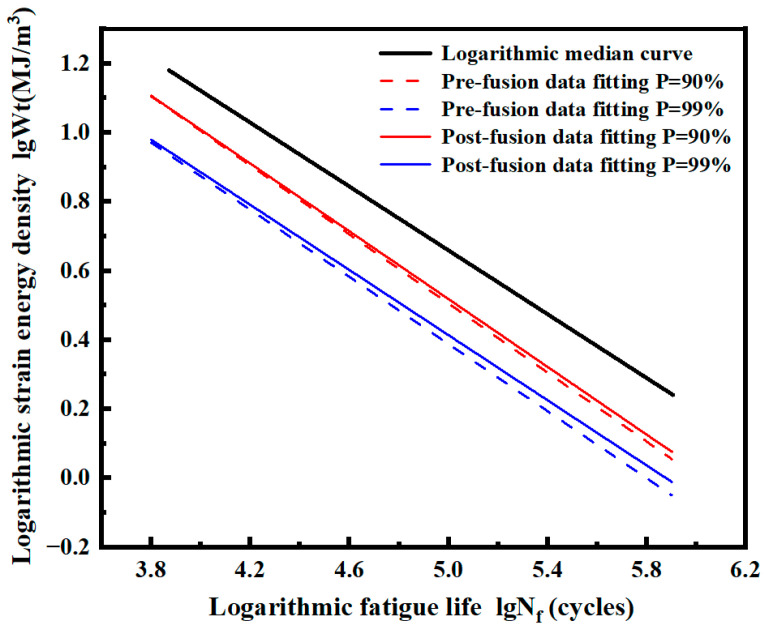
Median $W_{t}$-$N_{f}$ curve and logarithmic *P*-$W_{t}$-$N_{f}$ curve of smooth specimens before and after fusion.

The *P*-$W_{t}$-$N_{f}$ curves of smooth specimens before and after fusion in logarithmic coordinates are calculated, as shown in Figure 27. The results show that the fitted curve after fusion is above the curve before fusion under both probabilities *P*. The two curves are close at a high strain energy density level, while the deviation increases gradually at a low strain energy density level.

**Figure 28:**
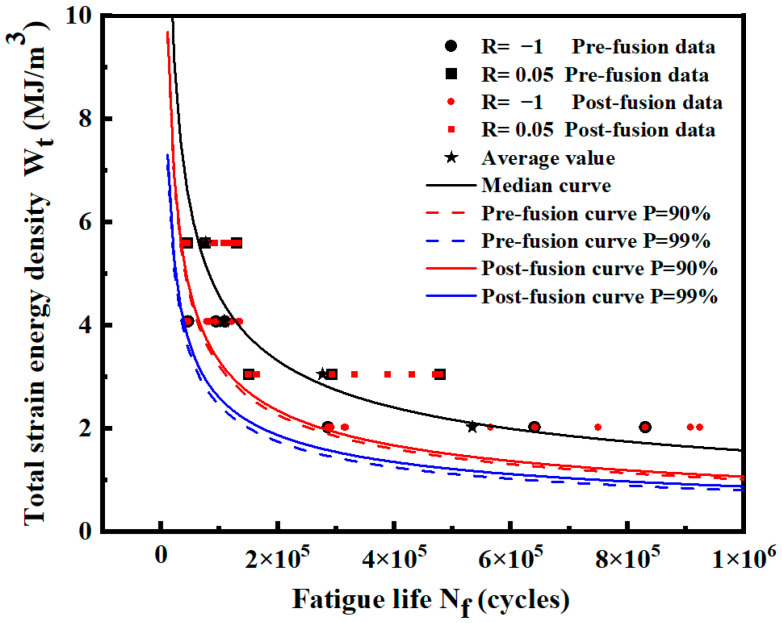
Data distribution and *P*-$W_{t}$-$N_{f}$ curves of smooth specimens before and after fusion.

Figure 28 shows the distribution of data and the *P*-$W_{t}$-$N_{f}$ curve before and after fusion. The expressions of each fitting curve are shown in Table 16. Most of the fused data are evenly distributed among the original data, which ensures that the mean fatigue life at the same strain energy density level after fusion remains unchanged, reduces the dispersion of low strain energy level data, and reduces the standard deviation.

The predicted lives of the two curves before and after fusion under different strain energy densities are listed in Table 16. The results show that the curve after fusion gradually deviates from the curve before fusion, and the predicted life of the curve after fusion is higher from high strain energy density to low strain energy density, which is similar to the overall data in Section 3.1. After fusion, the low strain energy density level with a smaller standard deviation is closer to the real overall data, and the life prediction is more accurate. Based on the *P*-$W_{t}$-$N_{f}$ curve given in Figure 28, the corresponding life can be obtained by calculating the strain energy density under different stress ratios.

For notched specimens, data fusion is also performed according to the test data in Table 5. The data before and after fusion are shown in Table 17, and the comparison of logarithmic mean and logarithmic standard deviation before and after fusion is shown in Table 18. The results show that after the fusion of different stress ratios, the average logarithmic life of each strain energy density level remains unchanged. In terms of standard deviation, after data fusion expands the sample data volume, the standard deviation between the data is reduced at the low strain energy density level (such as 0.807 MJ/m^3^). Compared with the pre-fusion data, the maximum reduction in the standard deviation of the post-fusion data is 38.5%. At the high strain energy density stage (such as 1.917 MJ/m^3^), the fatigue life of the notched specimens at fracture is small and relatively concentrated due to the large stress concentration coefficient. The standard deviation between the stages will be slightly increased after data fusion to expand the sample data. In the case of notched specimens, the data fusion method can not only expand the data but also reduce the dispersion of low-strain energy density level data.

**Figure 29:**
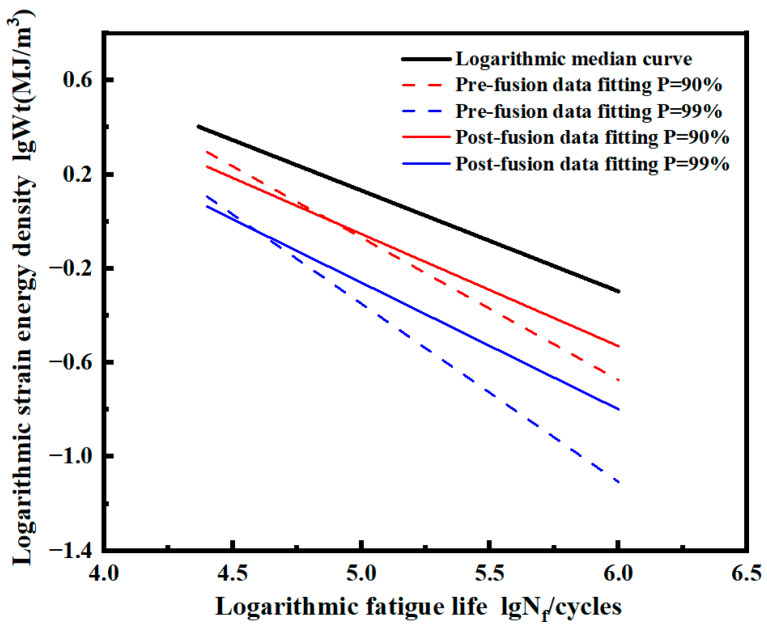
Median $W_{t}$-$N_{f}$ curve and logarithmic *P*-$W_{t}$-$N_{f}$ curve of notched specimens before and after fusion.

The *P*-$W_{t}$-$N_{f}$ curve of the pre-fusion data and post-fusion data of the notched specimen in logarithmic coordinates is calculated, as shown in Figure 29. Under both probabilities *P*, the fitted curve after fusion is above the curve before fusion. The two curves are close at a high strain energy density level, and the deviation increases at a low strain energy density level.

**Figure 30:**
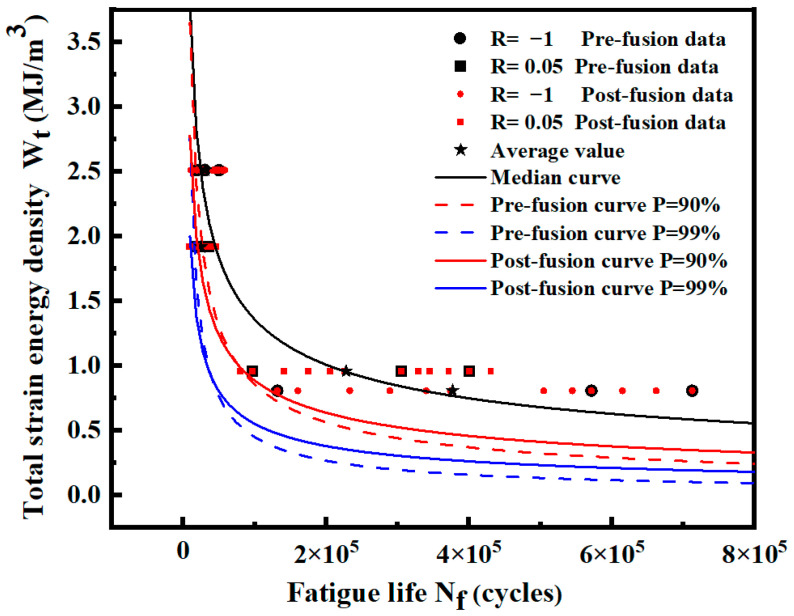
Data distribution and *P*-$W_{t}$-$N_{f}$ curves of notched specimens before and after fusion.

Figure 30 shows the distribution of data and the *P*-$W_{t}$-$N_{f}$ curve before and after fusion. The expression of the probability curve *P* = 90% and *P* = 99% before and after fusion is shown in Table 19. Most of the fused data are evenly distributed among the original data. At low strain energy density levels, the expansion of sample data reduces the standard deviation of each level and decreases the dispersion of data. At a high strain energy density level, the data dispersion will slightly increase after fusion.

The predicted lives of the two curves before and after fusion under different strain energy densities are listed in Table 19. The predicted life of the curve after fusion is larger at a low strain energy density level. With the change in data standard deviation, the curve before and after fusion gradually deviates from high-strain energy density to low-strain energy density. The predicted life of the curve after fusion gradually exceeds that of the curve before fusion. Therefore, the standard deviation is reduced at the low strain energy density level, which is closer to the real population data. A more accurate *P*-$W_{t}$-$N_{f}$ curve can be obtained by data fusion.

## 4. Conclusions

In this paper, to accurately evaluate the probabilistic characteristics of fatigue properties with small sample data under different stress ratios, a data fusion method for torsional fatigue life under different stress ratios was proposed based on the energy method. Numerical simulation was used to calculate the fatigue strain energy density. It was verified that the $W_{t}$-$N_{f}$ model under different stress ratios can be represented by the same. Combined with the data fusion method, we realized data fusion for fatigue life under different stress ratios. The following conclusions were obtained:

(1) Torsional fatigue tests were conducted, and test data were obtained. Based on numerical simulation, a method for the calculation of strain energy density was established. And the strain energy densities of specimens under different working conditions were calculated to provide a database for subsequent studies.
(2) The $W_{t}$-$N_{f}$ and *P*-$W_{t}$-$N_{f}$ prediction models under two different stress ratios were fitted. By error analysis, more than 85% of the data were distributed within the ±2 scatter bands, which verified the homogeneity of the models under different stress ratios.
(3) A data fusion method for fatigue life under different stress ratios was proposed. The data fusion method can expand small sample data, significantly reducing the standard deviation of medium and low strain energy density levels. Compared with the pre-fusion data, the standard deviation of the post-fusion data was reduced by a maximum of 21.5% for the smooth specimens and 38.5% for the notched specimens. The life prediction accuracy of the fused *P*-$W_{t}$-$N_{f}$ curves increased.

## Figures and Tables

**Table 1:** TC4 alloy material constants [39].

T (°C)	E (GPa)	ν	$n^{\prime }$
20	109	0.34	0.07

**Table 2:** Comparison between the calculated and reported strain energy densities [39].

TC4 Alloy, T = 20 °C, Kt = 1, R = −1				
Maximum Stress σ_*max*_/MPa	Fatigue Life $N_{f}$/Cycles	Reference Strain Energy Density $W_{t}$/(MJ/m^3^)	Calculated Strain Energy Density $W_{t}$/(MJ/m^3^)	Relative Error
780	4443	12	11.7581	−2.02%
730	6262	10.3	10.32782	0.27%
680	8725	8.85	8.79789	−0.59%
630	12,441	7.63	7.56113	−0.90%
580	18,028	6.46	6.49351	0.52%
520	28,447	5.21	5.10411	−2.03%

**Table 3:** Material parameters for the investigated alloy [42].

T (°C)	E (GPa)	ν	$n^{\prime }$
25	196.6	0.302	0.07

**Table 4:** Fatigue life and strain energy density calculation results for smooth specimens.

Stress Ratio R	Maximum Stress σ_*max*_/MPa	Logarithmic Median Life $N_{f}$/Cycles	Strain Energy Density $W_{t}$/(MJ/m^3^)
−1	700	7447	7.2057
−1	600	39,084	4.9239
−1	550	77,983	4.0795
−1	500	232,809	3.3485
−1	400	533,335	2.0273
−1	350	803,526	1.5393
0.05	1100	15,524	23.5288
0.05	1000	26,363	14.5014
0.05	900	32,885	6.6179
0.05	850	76,560	5.5978
0.05	800	177,419	4.5450
0.05	700	276,694	3.0503
0.05	650	322,833	2.4364
0.05	600	533,188	1.9197

**Table 5:** Fatigue life and strain energy density calculation results for notched specimens.

Stress Ratio R	Maximum Stress σ_*max*_/MPa	Logarithmic Median Life $N_{f}$/Cycles	Strain Energy Density $W_{t}$/(MJ/m^3^)
−1	500	27,356	3.0272
−1	450	44,030	2.6378
−1	400	30,654	2.5113
−1	350	38,493	2.1038
−1	300	85,066	1.5463
−1	250	376,897	0.8068
−1	200	1,000,000	0.5165
0.05	700	23,287	2.3784
0.05	650	24,578	1.9174
0.05	600	50,506	1.8471
0.05	550	47,405	1.3267
0.05	500	227,879	0.9575
0.05	450	228,355	0.7662
0.05	400	968,486	0.5294

**Table 6:** Comparison of different model expressions and predicted life of smooth specimens.

Model Type	Different Stress Ratio Conditions	Model Expression	R^2^ Value	Predicted Life of Different Strain Energy Densities/Cycles	
				5 MJ/m^3^	3 MJ/m^3^
Two-parameter model	R = −1	$W_{t}$ = 133.65($N_{f}$)^−0.315^	0.94	33,897	171,568
	R = 0.05	$W_{t}$ = 7793.28($N_{f}$)^−0.631^	0.92	114,769	257,875
	R = −1 and 0.05	$W_{t}$ = 932.65($N_{f}$)^−0.462^	0.85	82,232	248,446
Three-parameter model	R = −1	$W_{t}$ = 167.07($N_{f}$)^−0.335^	0.94	35,403	162,658
	R = 0.05	$W_{t}$ = 99797.05($N_{f}$)^−0.883^ + 1.10	0.91	98,205	221,731
	R = −1 and 0.05	$W_{t}$ = 40616.28($N_{f}$)^−0.825^ + 1.10	0.83	74,107	177,182

**Table 7:** Comparison of different model expressions and predicted life of notched specimens.

Model Type	Different Stress Ratio Conditions	Model Expression	R^2^ Value	Predicted Life of Different Strain Energy Densities/Cycles	
				2 MJ/m^3^	1 MJ/m^3^
Two-parameter model	R = −1	$W_{t}$ = 355.959($N_{f}$)^−0.474^	0.97	55,926	241,379
	R = 0.05	$W_{t}$ = 95.649($N_{f}$)^−0.380^	0.94	26,311	163,049
	R = −1 and 0.05	$W_{t}$ = 189.54($N_{f}$)^−0.429^	0.91	40,516	203,857
Three-parameter model	R = −1	$W_{t}$ = 1472.68($N_{f}$)^−0.617^ + 0.23	0.97	54,050	208,289
	R = 0.05	$W_{t}$ = 942.00($N_{f}$)^−0.614^ + 0.33	0.94	30,269	133,967
	R = −1 and 0.05	$W_{t}$ = 4631.96($N_{f}$)^−0.747^ + 0.37	0.89	42,001	149,946

**Table 8:** The fitting model of the smooth specimen under different conditions.

Different Stress Ratio Conditions	Probability Level	Model Expression
R = −1	P = 90%	$W_{t}$ = 162.31($N_{f}$)^−0.347^
	P = 99%	$W_{t}$ = 178.43($N_{f}$)^−0.368^
R = 0.05	P = 90%	$W_{t}$ = 4026.15($N_{f}$)^−0.622^
	P = 99%	$W_{t}$ = 1440.39($N_{f}$)^−0.563^
R = −1 and 0.05	P = 90%	$W_{t}$ = 1008.63($N_{f}$)^−0.500^
	P = 99%	$W_{t}$ = 657.25($N_{f}$)^−0.486^

**Table 9:** The fitting model of the notched specimen under different conditions.

Different Stress Ratio Conditions	Probability Level	Model Expression
R = −1	P = 90%	$W_{t}$ = 2476.45 ($N_{f}$)^−0.694^
	P = 99%	$W_{t}$ = 10476.59($N_{f}$)^−0.894^
R = 0.05	P = 90%	$W_{t}$ = 306.27($N_{f}$)^−0.511^
	P = 99%	$W_{t}$ = 983.08($N_{f}$)^−0.657^
R = −1 and 0.05	P = 90%	$W_{t}$ = 911.55($N_{f}$)^−0.605^
	P = 99%	$W_{t}$ = 2775.81($N_{f}$)^−0.759^

**Table 10:** Fatigue test data before fusion.

Strain Energy Density/(MJ/m^3^)	Logarithmic Fatigue Life /Cycles
6.639	4.624, 4.699, 4.678, 4.713, 4.768
4.652	4.914, 4.992, 5.014, 5.065, 5.127
2.987	5.472, 5.532, 5.560, 5.607, 5.677

**Table 11:** Fatigue test data after fusion.

Strain Energy Density/(MJ/m^3^)	Logarithmic Fatigue Life /Cycles
6.639	4.624, 4.699, 4.678, 4.713, 4.768, 4.593, 4.653, 4.681, 4.727, 4.798, 4.582, 4.660, 4.682, 4.734, 4.795
4.652	4.914, 4.992, 5.014, 5.065, 5.127, 4.925, 4.985, 5.013, 5.059, 5.130, 4.956, 5.001, 5.010, 5.045, 5.100
2.987	5.472, 5.532, 5.560, 5.607, 5.677, 5.461, 5.539, 5.562, 5.613, 5.674, 5.503, 5.549, 5.558, 5.592, 5.647

**Table 12:** Comparison of mean and standard deviation for pre-fusion, post-fusion, and overall data.

Strain Energy Density/(MJ/m^3^)	Pre-Fusion Fatigue Data $N_{f}$/Cycles		Post-Fusion Fatigue Data $N_{f}$/Cycles		$\mathrm{Overall}\;\mathrm{Large}\;\mathrm{Sample}\;\mathrm{Data}\;N_{f}$/Cycles	
	Logarithmic Mean	Logarithmic Standard Deviation	Logarithmic Mean	Logarithmic Standard Deviation	Logarithmic Mean	Logarithmic Standard Deviation
6.639	4.69046	0.053750	4.69046	0.065993	4.73836	0.067467
4.652	5.02218	0.079866	5.02218	0.065994	5.08858	0.089017
2.987	5.56984	0.077304	5.56984	0.065995	5.63849	0.094436

**Table 13:** Expressions of each probability *P*-$W_{t}$-$N_{f}$ curve under pre-fusion, post-fusion, and overall data.

Data Type	Probability Level	Model Expression	Predicted Life of Different Strain Energy Densities and Relative Error Based on Overall Data					
			6.639 MJ/m^3^	Relative Error	4.652 MJ/m^3^	Relative Error	2.987 MJ/m^3^	Relative Error
Pre-fusion data	P = 50%	$W_{t}$ = 438.94($N_{f}$)^−0.390^	46,499	11.48%	115,745	13.15%	360,451	15.19%
	P = 99%	$W_{t}$ = 476.12($N_{f}$)^−0.411^	32,725	6.99%	77,750	7.58%	228,473	8.31%
Post-fusion data	P = 50%	$W_{t}$ = 438.94($N_{f}$)^−0.390^	46,499	11.48%	115,745	13.15%	360,451	15.19%
	P = 99%	$W_{t}$ = 381.36($N_{f}$)^−0.390^	32,423	7.85%	80,707	4.07%	251,337	0.87%
Overall data	P = 50%	$W_{t}$ = 421.98($N_{f}$)^−0.382^	52,528	-	133,274	-	425,031	-
	P = 99%	$W_{t}$ = 475.35($N_{f}$)^−0.408^	35,184	-	84,127	-	249,181	-

**Table 14:** Fatigue life data of smooth specimens under different stress ratios before and after fusion.

Stress Ratio R	Strain Energy Density $W_{t}$/(MJ/m^3^)	Logarithmic Fatigue Life before Fusion $N_{f}$/Cycles	Logarithmic Fatigue Life after Fusion $N_{f}$/Cycles
−1	4.079	4.971, 5.039, 4.664	4.971, 5.039, 4.664, 4.621, 4.971, 5.083, 4.892, 4.661, 5.122, 5.130, 4.629, 4.916
	2.027	5.456, 5.806, 5.919	5.456, 5.806, 5.919, 5.807, 5.875, 5.500, 5.727, 5.497, 5.958, 5.965, 5.465, 5.752
0.05	5.598	4.884, 4.653, 5.114	4.884, 4.653, 5.114, 4.964, 5.031, 4.656, 4.613, 4.963, 5.076, 5.122, 4.621, 4.908
	3.050	5.681, 5.180, 5.467	5.681, 5.180, 5.467, 5.522, 5.590, 5.215, 5.171, 5.521, 5.634, 5.442, 5.212, 5.673

**Table 15:** Comparison of mean and standard deviation of smooth specimens under different stress ratios before and after fusion.

Stress Ratio R	Strain Energy Density $W_{t}$/(MJ/m^3^)	Pre-Fusion Data *N_f_*/Cycles		Post-Fusion Data *N_f_*/Cycles	
		Logarithmic Mean	Logarithmic Standard Deviation	Logarithmic Mean	Logarithmic Standard Deviation
−1	4.079	4.88153	0.1997664	4.88153	0.1975206
	2.027	5.71650	0.2414045	5.71650	0.1975221
0.05	5.598	4.89483	0.2306621	4.88483	0.1975220
	3.050	5.44238	0.2513805	5.44238	0.1975213

**Table 16:** Expressions of probability *P*-$W_{t}$-$N_{f}$ curves of smooth specimens before and after fusion.

Data Type	Probability Level	Model Expression	Predicted Life of Different Strain $\mathrm{Energy}\;\mathrm{Densities}\;N_{f}$/Cycles			
			5.598 MJ/m^3^	4.079 MJ/m^3^	3.050 MJ/m^3^	2.027 MJ/m^3^
Pre-fusion data	P = 90%	$W_{t}$ = 1008.63($N_{f}$)^−0.500^	32,464	61,144	109,361	247,603
	P = 99%	$W_{t}$ = 657.25($N_{f}$)^−0.486^	18,140	34,795	63,285	146,694
Post-fusion data	P = 90%	$W_{t}$ = 934.74($N_{f}$)^−0.491^	33,636	64,091	115,860	266,275
	P = 99%	$W_{t}$ = 590.47($N_{f}$)^−0.471^	19,745	38,668	71,682	170,668

**Table 17:** Fatigue life data of notched specimens under different stress ratios before and after fusion.

Stress Ratio R	Strain Energy Density $W_{t}$/(MJ/m^3^)	Logarithmic Fatigue Life before Fusion $N_{f}$/Cycles	Logarithmic Fatigue Life after Fusion $N_{f}$/Cycles
−1	2.511	4.277, 4.483, 4.699	4.277, 4.483, 4.699, 4.667, 4.763, 4.029, 4.646, 4.442, 4.371, 4.115, 4.731, 4.613
	0.807	5.757, 5.853, 5.119	5.757, 5.853, 5.119, 5.367, 5.573, 5.789, 5.736, 5.531, 5.461, 5.205, 5.821, 5.703
0.05	1.917	4.550, 4.346, 4.275	4.550, 4.346, 4.275, 4.182, 4.387, 4.603, 4.571, 4.667, 3.933, 4.019, 4.635, 4.517
	0.957	4.986, 5.602, 5.484	4.986, 5.602, 5.484, 5.149, 5.354, 5.570, 5.538, 5.634, 4.901, 5.518, 5.313, 5.243

**Table 18:** Comparison of mean and standard deviation of notched specimens under different stress ratios before and after fusion.

Stress Ratio R	Strain Energy Density $W_{t}$/(MJ/m^3^)	Pre-Fusion Data *N_f_*/Cycles		Post-Fusion Data *N_f_*/Cycles	
		Logarithmic Mean	Logarithmic Standard Deviation	Logarithmic Mean	Logarithmic Standard Deviation
−1	2.511	4.48648	0.2106861	4.47548	0.2451951
	0.807	5.57622	0.3987908	5.57735	0.2451965
0.05	1.917	4.39054	0.1428186	4.38061	0.2451956
	0.957	5.35770	0.3268738	5.35770	0.2451963

**Table 19:** Expressions of probability *P*-$W_{t}$-$N_{f}$ curves of notched specimens before and after fusion.

Data Type	Probability Level	Model Expression	Predicted Life of Different Strain $\mathrm{Energy}\;\mathrm{Densities}\;N_{f}$/Cycles			
			2.511 MJ/m^3^	1.917 MJ/m^3^	0.957 MJ/m^3^	0.807 MJ/m^3^
Pre-fusion data	P = 90%	$W_{t}$ = 911.55($N_{f}$)^−0.606^	16,762	26,167	82,343	109,094
	P = 99%	$W_{t}$ = 2775.81($N_{f}$)^−0.759^	10,231	14,601	36,467	45,650
Post-fusion data	P = 90%	$W_{t}$ = 214.78($N_{f}$)^−0.477^	11,236	19,787	84,897	121,370
	P = 99%	$W_{t}$ = 271.79($N_{f}$)^−0.539^	5948	9814	35,614	48,863

## Data Availability

The original contributions presented in the study are included in the article, further inquiries can be directed to the corresponding author.

## Nomenclature

S	fatigue stress	$n^{\prime }$	cyclic strain hardening index
N	fatigue life	$K^{\prime }$	cyclic strength coefficient
P	survival rate	T	temperature
$W_{t}$	strain energy density	Kt	stress concentration factor
${\Delta W}_{e}$	elastic strain energy density	R	stress ratio
${\Delta W}_{p}$	plastic strain energy density	ν	Poisson ratio
ε	strain	$x_{a}$	average fatigue life
σ	stress	$x_{p}$	probabilistic fatigue life
$\sigma_{max}$	Maximum stress	$k_{\left(p,\gamma ,\nu \right)}$	one-side tolerance factor
Δε	strain range	$n_{ji}$	fatigue life of specimen i at strain energy density level j
Δσ	stress range	$p\left(n_{ji}\right)$	probability of life being less than $n_{ji}$ at strain energy density level j
$\varepsilon_{p}$	plastic strain	$\mu_{i}$	Mean fatigue life at strain energy density level i
$\varepsilon_{e}$	elastic strain	$\sigma_{i}$	standard deviation at strain energy density level i
E	elastic modulus		
